# Live-cell imaging of nuclear–chromosomal dynamics in bovine *in vitro* fertilised embryos

**DOI:** 10.1038/s41598-018-25698-w

**Published:** 2018-05-10

**Authors:** Tatsuma Yao, Rie Suzuki, Natsuki Furuta, Yuka Suzuki, Kyoko Kabe, Mikiko Tokoro, Atsushi Sugawara, Akira Yajima, Tomohiro Nagasawa, Satoko Matoba, Kazuo Yamagata, Satoshi Sugimura

**Affiliations:** 1 Faculty of Biology-Oriented Science and Technology (BOST), Kindai University, Wakayama, Japan; 2 Research and Development Center, Fuso Pharmaceutical Industries, Ltd., Osaka, Japan; 3 Asada Institute for Reproductive Medicine, Asada Ladies Clinic, Aichi, Japan; 4 Department of Biological Production, Tokyo University of Agriculture and Technology, Tokyo, Japan; 5 Institute for Biogenesis Research, University of Hawaii Medical School, Honolulu, Hawaii USA; 6 Division of Animal Breeding and Reproduction Research, Institute of Livestock and Grassland Science, NARO (NILGS), Ibaraki, Japan

## Abstract

Nuclear/chromosomal integrity is an important prerequisite for the assessment of embryo quality in artificial reproductive technology. However, lipid-rich dark cytoplasm in bovine embryos prevents its observation by visible light microscopy. We performed live-cell imaging using confocal laser microscopy that allowed long-term imaging of nuclear/chromosomal dynamics in bovine *in vitro* fertilised (IVF) embryos. We analysed the relationship between nuclear/chromosomal aberrations and *in vitro* embryonic development and morphological blastocyst quality. Three-dimensional live-cell imaging of 369 embryos injected with mRNA encoding histone H2B-mCherry and enhanced green fluorescent protein (EGFP)-α-tubulin was performed from single-cell to blastocyst stage for eight days; 17.9% reached the blastocyst stage. Abnormalities in the number of pronuclei (PN), chromosomal segregation, cytokinesis, and blastomere number at first cleavage were observed at frequencies of 48.0%, 30.6%, 8.1%, and 22.2%, respectively, and 13.0%, 6.2%, 3.3%, and 13.4%, respectively, for abnormal embryos developed into blastocysts. A multivariate analysis showed that abnormal chromosome segregation (ACS) and multiple PN correlated with delayed timing and abnormal blastomere number at first cleavage, respectively. In morphologically transferrable blastocysts, 30–40% of embryos underwent ACS and had abnormal PN. Live-cell imaging may be useful for analysing the association between nuclear/chromosomal dynamics and embryonic development in bovine embryos.

## Introduction

Due to global advances in genomic selection and gene-editing in recent years, *in vitro* fertilised (IVF) embryo transfer has become an important innovation in the agricultural sectors, such as in cattle production. Approximately half of all bovine embryos produced worldwide were derived from IVF^1^. However, the pregnancy success rate of IVF embryos transplanted into recipients remains low (30–40%), which is inferior to the pregnancy success rate of transplanting *in vivo* derived embryos^2^. To increase the success of pregnancy, key technological issues affecting the *in vitro* production of embryos and the assessment of viable embryos must be addressed. This is also true for human artificial reproductive technology (ART)^3^. Generally, assessment of bovine embryo quality is performed by morphological grading on days 7 to 8 post-insemination, as recommended by the International Embryo Transfer Society (IETS)^4,5^, but the pregnancy success rate of embryos judged as transferable (Code 1 or Code 2) is only 30–50%^6,7^.

In human ART, assessment of embryo quality using time-lapse cinematography with a visible light microscope has recently become a popular technology. Morphokinetic parameters such as the number of pronuclei (PN) or nuclei, timing of cleavage, and number of blastomeres, are used as potential indicators that may improve the success of ART^3,8^. Furthermore, it has been reported that ART success is improved by comprehensive chromosomal screening, using techniques such as array comparative genomic hybridization (CGH), quantitative single nucleotide polymorphism (SNP) arrays, and next-generation sequencing^8^.

Using time-lapse cinematography analysis in cattle, we recently found that morphokinetic indicators (MKIs) such as timing, number of blastomeres at first cleavage, and number of blastomeres at onset of the lag-phase, are useful markers for evaluating embryo viability after transfer to a recipient^7,9^. Therefore, using MKIs may be a better assessment method than using IETS morphological grading, in terms of reliability and objectivity of evaluation of bovine IVF embryos^9^. However, biological information obtained by time-lapse cinematography is limited and lipid-rich dark cytoplasm prevents PN/nuclear observation in bovine IVF embryos.

Techniques have been developed to visualise nuclear/chromosomal dynamics in mice by long-term live-cell imaging^10,11^. This consists of mRNA injection and time-lapse fluorescence confocal microscopy. Live-cell imaging revealed that almost all mouse embryos with abnormal chromosome segregation (ACS) during the first mitosis have no viability after transfer^11^. Here, we performed live-cell imaging in bovine IVF embryos. To evaluate the relationship between nuclear/chromosomal abnormalities and embryonic development and morphological blastocyst quality, we injected zygotes with mRNA encoding α-tubulin tagged with enhanced green fluorescent protein (EGFP) as a microtubule marker and histone H2B fused with mCherry as a chromatin marker. This method enabled the evaluation of nuclear/chromosomal integrity in the presence of dark cytoplasm.

## Results

### Impact of live-cell imaging on *in vitro* bovine embryo development

To evaluate the impact of live-cell imaging on embryonic development, we first investigated the effect of mRNA injection. In cultivation using a conventional incubator, there was no effect of mRNA probe injection on blastocyst development in bovine zygotes (Table 1). Subsequently, to determine optimal imaging conditions, embryos injected with mRNA probes of histone H2B-mCherry and EGFP-α-tubulin were exposed to a laser for various durations at different wavelengths. The best blastocyst yield was obtained when the embryos were exposed for 50 msec at 488 nm and 100 msec at 561 nm (Table 1). This developmental competence was not inferior to that of embryos that were not exposed to live-cell imaging (*P* > 0.05). In a preliminary assessment of embryo transfer, pregnancy with foetal heartbeat was diagnosed at days 31 and 45 (Supplementary Movie S1). This optimised imaging condition was used in subsequent analysis.

**Table 1:** Effects of live-cell imaging on bovine embryo development.

Incubator	Exposure time (msec)		Z slices	Sham injection	mRNA injection	No. of blastocysts/ cultured (%)
	488 nm	561 nm				
Conventional incubator (APM-30DR)	−	−	−	−	−	18/60 (31.7%)
	−	−	−	+	−	22/68 (32.4%)
Imaging system (CV1000)	−	−	−	−	+	15/48 (31.3%)
	150	150	31	−	+	7/42 (16.7%)
	100	100	31	−	+	3/24 (12.5%)
	50	100	31	−	+	19/52 (36.5%)

### Relationship between abnormal fertilisation and first cleavage and subsequent *in vitro* developmental competence

**Figure 1:**
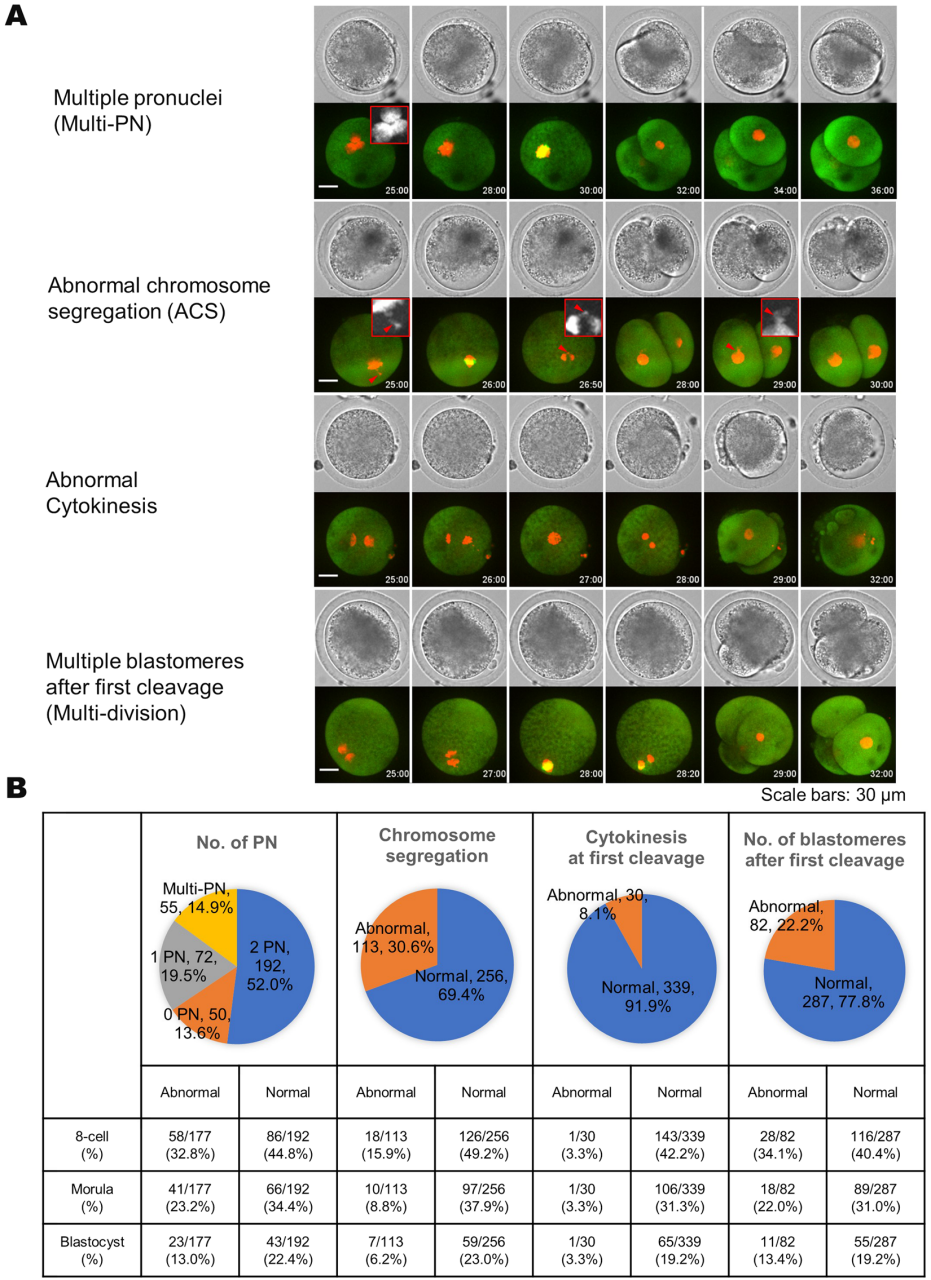
Correlation between abnormal first cleavage and subsequent *in vitro* development. Pronuclear (PN) number was set to 0 (0 PN), 1 (1 PN), 2 (2 PN), and three or more pronuclei (multi-PN). 0 PN, 1 PN, and multi-PN were defined as abnormal number of pronuclei. Abnormal chromosome segregation (ACS) was defined as the misalignment of a portion of the chromosomes with the metaphase plate, and/or the lagging chromosomes at the anaphase stage. Red arrows show micronuclei-like structures. Abnormal cytokinesis was defined as the occurrence of chromosome segregation, in which the cleavage plane did not appear completely and blastomere number did not increase after chromosome segregation. Abnormal number of blastomeres was defined as three or more blastomeres observed at the end of first cleavage (multi-division). Scale bars are 30 μm (**A**). Frequency and subsequent developmental competence to 8-cell, morulae, and blastocyst stages in inseminated oocytes with normal and abnormal first cleavage were calculated (**B**).

The developmental competence of the populations of embryos with abnormalities in the number of PN, chromosome segregation, cytokinesis at first cleavage, and number of blastomeres after first cleavage was analysed (Fig. 1B). Forty-eight percent of embryos (177/369) had an abnormal number of PN [0 PN = 13.6%, 1 PN = 19.5%, and multi-PN ( ≥ 3) = 14.9%]; 13.0% of them developed to the blastocyst stage. This value was significantly lower than that of embryos with a normal number of PN, 22.4% (43/192) of which developed into blastocysts (*P* = 0.019). ACS occurred in 30.6% of embryos, most of which stopped developing before the eight-cell stage, with only 6.2% of them developing into blastocysts. Eight point one percent of embryos exhibited abnormal cytokinesis at first cleavage and almost all of these embryos stopped developing before the eight-cell stage (Fig. 1B). Treatment with okadaic acid during oocyte maturation, which induces cytokinesis defects via inhibition of protein phosphatase type 1 and type 2^12^, increased abnormal cytokinesis and also inhibited embryonic development from cell-stages two to eight (Supplementary Tables S1 and S2). At the end of first cleavage, 22.2% of embryos had an abnormal number of blastomeres and 13.4% of these developed into blastocysts. This was comparable to blastocyst development of embryos with normal blastomere numbers (19.2%, *P* = 0.231). Multivariate statistical analysis indicated that blastocyst development was related to the presence of one PN and ACS at first cleavage (Supplementary Table S3). These results suggested a relationship between abnormal first cleavage and inferior embryo development.

### Factors relevant to timing and blastomere number at first cleavage and blastomere number at lag-phase

**Figure 2:**
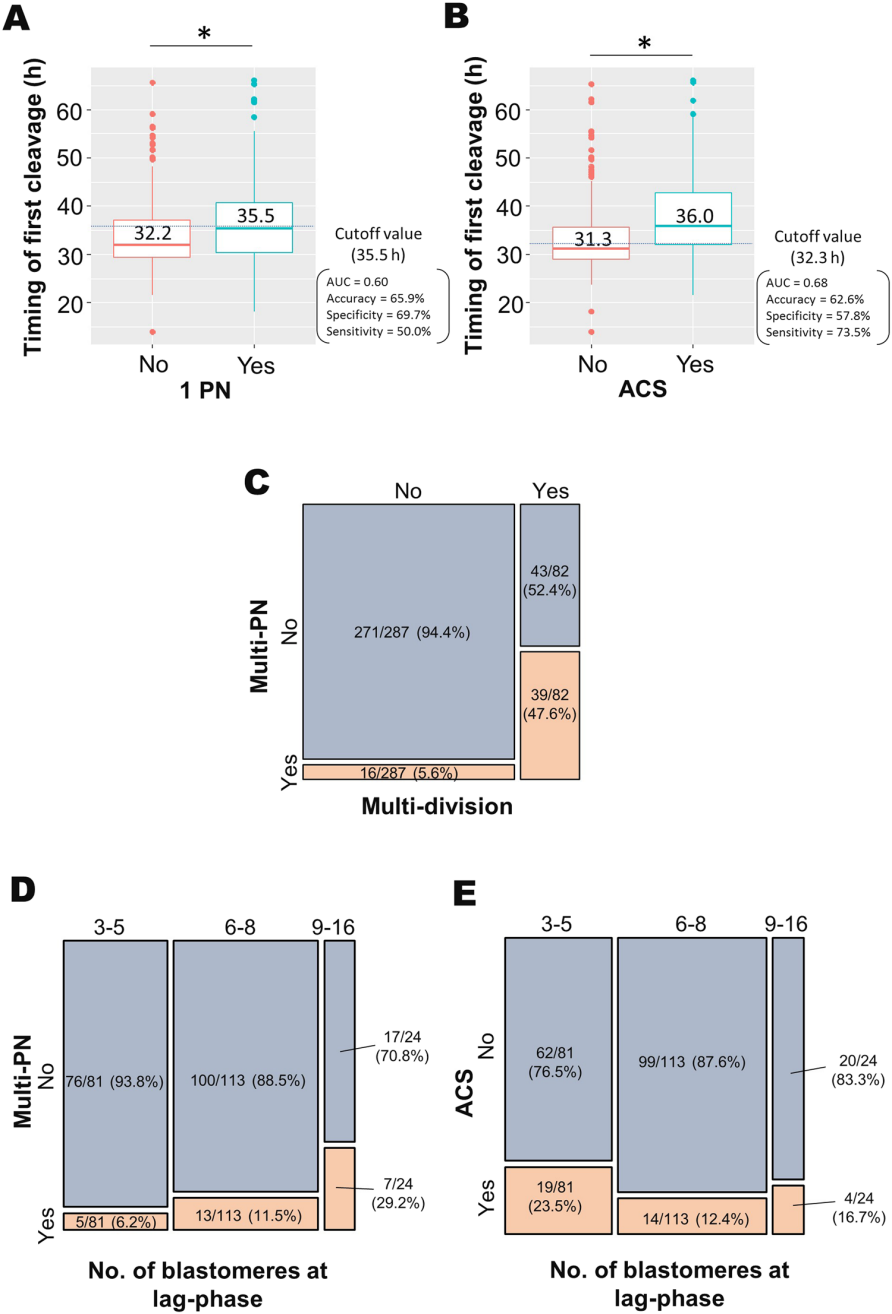
Relationship between morphokinetic indicators and abnormalities of pronuclear number and chromosome segregation. Timing of first cleavage after insemination in embryos with or without one pronucleus (1PN) and abnormal chromosome segregation (ACS) was shown in (**A**) and (**B**), respectively. Asterisks indicate a significant difference between groups based on Wilcoxon rank sum test (*P = 0.008, *P* < 0.001). A hierarchical relationship between more than three blastomeres (multi-division) and three or more pronuclei (multi-PN) is shown by a mosaic plot (**C**). Hierarchical relationships between the number of blastomeres at lag-phase and multi-PN (**D**) and ACS (**E**) are shown by mosaic plots.

A multivariate statistical analysis indicated that the timing of the first cleavage was related to embryos with one PN and ACS (Supplementary Table S4). The timing of the first cleavage in oocytes with one PN (Fig. 2A) or ACS (Fig. 2B) was significantly slower compared to that of oocytes without these abnormalities (*P* = 0.008 and *P* < 0.001, respectively). Multi-PN number was related to an abnormal number of blastomeres, which was defined as three or more blastomeres observed at the end of the first cleavage (multi-division) (Supplementary Table S5). As shown in Fig. 2C, 47.6% of the embryos with multi-division had multi-PN, whereas only 5.6% with normal blastomeres had multi-PN. Interestingly, only two PN were involved in syngamy, and these nuclei formed mitotic spindles at each position and segregated in multi-PN embryos, undergoing multi-division (Supplementary Movie S2). The blastomere number at the onset of lag-phase (identified as a temporary developmental arrest during the fourth or fifth cell cycle) was related to the multi-PN number and ACS (Supplementary Table S6). Of embryos with three to five and six to eight blastomeres at lag-phase, 6.2% and 11.5%, respectively, had multi-PN, while 29.2% with nine to sixteen blastomeres had multi-PN (Fig. 2D). On the other hand, of the embryos with six to eight and nine to sixteen blastomeres at lag-phase, 12.4% and 16.7%, respectively, underwent ACS at first cleavage, while 23.5% with three to five blastomeres underwent ACS (Fig. 2E). These results demonstrate that morphokinetic indicators were strongly influenced by PN number and/or chromosome segregation.

### Relationship between morphologically graded embryo quality and nuclear/chromosomal abnormalities

**Figure 3:**
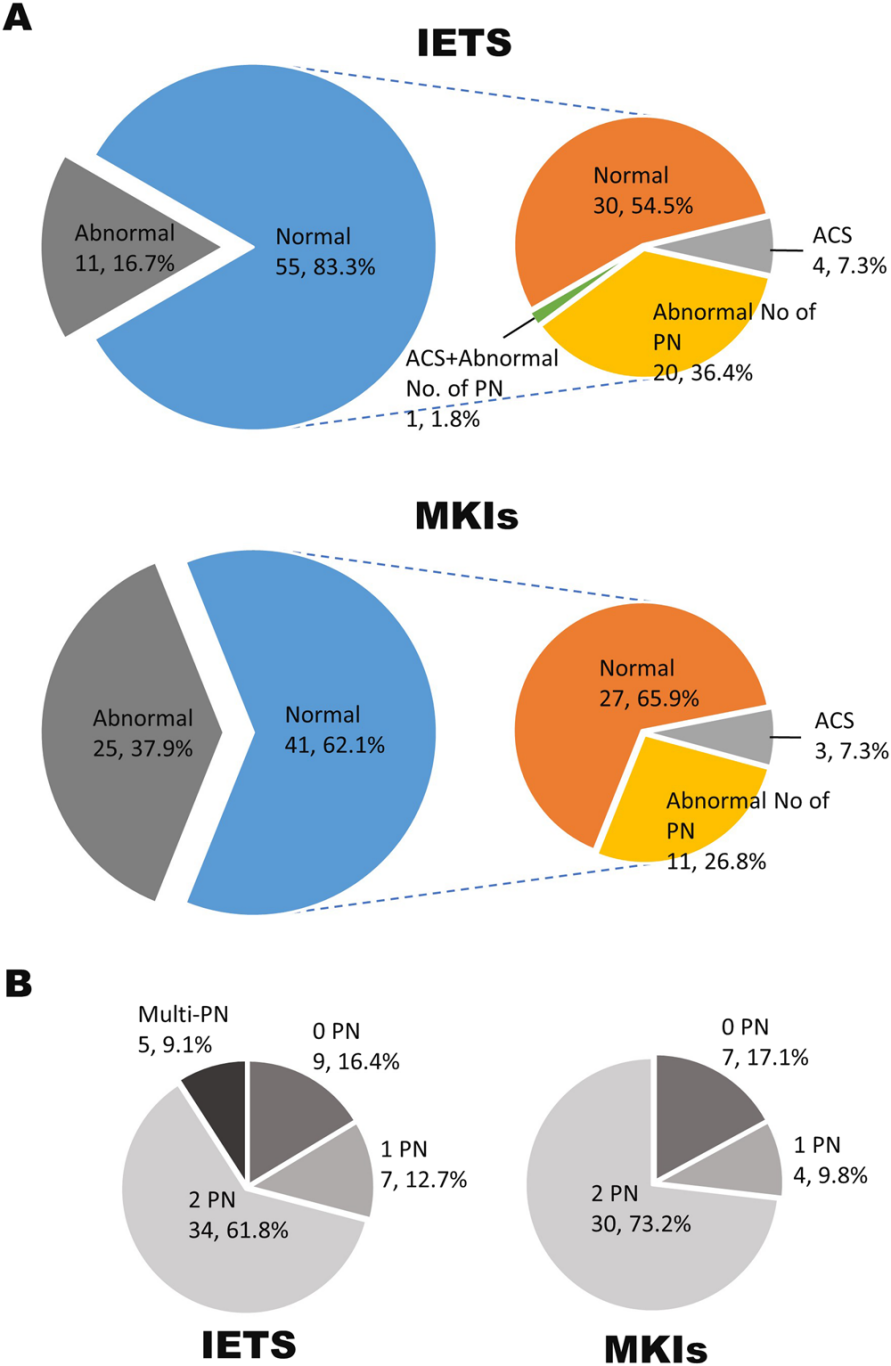
Population of blastocysts with abnormal pronuclear number or ACS that were graded as transferable (normal) by criteria of the International Embryo Transfer Society (IETS) and morphokinetic indicators (MKIs). According to IETS criteria, code 1 and 2 were defined as normal. Using MKIs, blastocysts derived from embryos that accomplished the first cleavage within 32.3 h post-insemination, 2 blastomeres at the end of first cleavage, and 6–8 blastomeres at the onset of lag-phase were defined as normal. The pie chart in the right panel shows the populations of blastocysts with an abnormal pronuclear number or ACS in blastocysts judged as morphologically normal (**A**). The proportion of blastocysts derived from zygotes with no pronucleus (0 PN), one pronucleus (1 PN), two pronuclei (2 PN), and more than three pronuclei (multi-PN) were graded as normal by IETS criteria or MKIs (**B**).

We examined the incidence of nuclear/chromosomal abnormalities in blastocysts graded as morphologically transferable. According to IETS criteria, the populations of blastocysts that exhibited ACS, abnormal number of PN, and both ACS and abnormal number of PN were 7.3%, 36.4%, and 1.8%, respectively (Fig. 3A). On the other hand, in blastocysts assessed by MKIs, 7.3% and 26.8% exhibited ACS and abnormal number of PN, respectively (Fig. 3A). Interestingly, IETS criteria indicated that 9.1% of blastocysts had multi-PN, whereas assessment by MKIs indicated that no multi-PN blastocysts were observed (Fig. 3B). Therefore, blastocysts that were graded as morphologically normal according to IETS criteria and MKIs may have nuclear/chromosomal abnormalities. However, embryo quality assessment using morphokinetic indicators may reduce the risk of selecting embryos with multi-PN.

## Discussion

In this study, we succeeded in performing non-invasive long-term live-cell imaging of bovine IVF embryos with fluorescence confocal laser microscopy. This technique allowed visualisation of the nuclear/chromosomal dynamics of bovine embryos for eight days, and determination of various biological factors involved in the relationship between nuclear/chromosomal abnormalities and subsequent *in vitro* embryonic development and morphological embryo quality.

In human ART, the number of PN is the most important prerequisite for predicting the developmental competence of embryos. However, the lipid-rich dark cytoplasm of bovine embryos has inhibited observation of PN. In the present study, we observed that embryos with an abnormal number of PN had impaired embryo development compared with embryos with two PN. The blastocyst formation rates of oocytes with zero, one, two, and multi-PN were 18.0% (9/50), 9.7% (7/72), 22.4% (43/192), and 12.7% (7/55), respectively (Supplementary Table S7). The lower developmental competence of embryos with an abnormal number of PN, such as one PN and multi-PN, was consistent with that reported in a previous human study^13^. Embryos with an abnormal number of PN may be derived from *in vitro* maturation (IVM)/*in vitro* fertilisation (IVF) failure^14,15^. Further improvement of IVM and IVF technologies will be required to prevent deficient cytoplasmic maturation and abnormal fertilisation^16–18^.

ACS during *in vitro* embryonic development was demonstrated as a promising indicator of embryo viability in mouse ART^11,19^. When okadaic acid induced severe ACS in bovine IVF embryos, subsequent development decreased (Supplementary Tables S1 and S2, Supplementary Fig. S1, and Supplementary Movie S3). ACS was also observed during first cleavage in embryos under normal bovine IVF conditions, which exhibited lower blastocyst competence as well; however, ACS incidence (30.6%) was higher than that of mouse IVF embryos (1.7–3.5%)^11^. In mouse studies, ACS was caused by double-strand DNA breaks in the sperm genome^11^; the incidence rate was increased by exposing the sperm to freeze-thaw cycles^11^. In the present study, frozen semen was used for IVF, which may be a contributing factor to the high incidence of ACS^11^.

It has been well documented that delayed timing of first cleavage is involved in blastocyst formation and pregnancy success^7,9,20,21^. We observed a relationship between delayed timing of first cleavage and ACS. In a similar study using a pig embryo model, there was a correlation between the presence of double-strand DNA breaks and delayed embryo cleavage and decreased blastocyst formation^22^. In somatic cells, micronuclei, often observed during ACS, are formed due to DNA damage, including double-strand DNA breaks, and cause delayed and prolonged mitosis, which is regulated by the spindle assembly check point (SAC)^23^ for DNA repair. Hence, the delayed timing of the first cleavage may be a result of SAC activation by DNA damage pre- or post-fertilisation.

A previous study reported that embryos which cleaved directly into three to four cells (multi-division) at first cleavage have a high incidence of chromosomal abnormalities and a low viability after transfer^7,9,24^. In the present study, we confirmed that a multi-PN number was involved in multi-division. A study of human embryos also revealed that most tri-pronuclei cleaved directly into three cells^24^. Approximately half of the instances of multi-division were in multi-PN embryos, whereas multi-PN were scarcely observed in embryos with two blastomeres. Furthermore, in embryos that reached the blastocyst stage, there were no multi-PN observed in embryos with two blastomeres (Supplementary Fig. S2). Thus, the number of blastomeres at first cleavage may be useful for predicting the occurrence of multi-PN.

The lag-phase, which occurs in the fourth or fifth cell cycle in which the longer Gap 2 phase is inserted, corresponds to embryonic genome activation in cattle. Previously, we showed that a small number of blastomeres at the lag-phase was related to the higher incidence of apoptosis in blastocysts^9^. Live-cell imaging revealed a relationship between ACS and a low blastomere number at the lag-phase. It has been reported that DNA damage, such as double-strand breaks, may cause ACS^11^ and induce apoptosis in embryos^25^, which could be a reason for the high incidence of apoptosis observed.

In this study, nuclear/chromosomal abnormalities, such as ACS and abnormal number of PN, were observed in embryos that were graded as morphologically transferable, regardless of IETS criteria and MKIs. Thus, it may be difficult to judge nuclear/chromosomal abnormalities based on morphological evaluation. Indeed, karyotyping of blastocysts revealed that 14.3% (2/14) of *in vivo* embryos and 12.5% (1/8) of embryos selected by live-cell imaging contained mixoploids, whereas embryos selected by IETS and MKIs contained mixoploids at 42.9% (12/28) and 27.6% (8/29), respectively (Supplementary Fig. S3). Interestingly, since multi-PN embryos were not included in the MKI-selected embryos, monitoring the embryo development with time-lapse cinematography instead of the morphological “snapshot” evaluation used in accordance with IETS criteria may reduce the risk of selecting embryos with multi-pronuclei, which are clinically discarded from transferrable embryos in human ART^13^.

In conclusion, this live-cell imaging technique could be useful for analysing the association between nuclear/chromosomal dynamics and embryo development in bovine embryos whose nuclei/pronuclei are not observable by visible light microscopy.

## Materials and Methods

### Ethics statement

This study was approved by the Ethics Committee for the Care and Use of Experimental Animals at Tokyo University of Agriculture and Technology, located in Tokyo, and the NARO Institute of Livestock and Grassland Science Animal Care Committee in Tsukuba, Japan. All animals received humane care according to guideline numbers 6, 22, and 105 of the Japanese Guidelines for Animal Care and Use.

### Chemicals

Unless specified, all chemicals were purchased from Sigma-Aldrich (St Louis, MO, USA).

### Oocyte collection

Ovaries from Japanese Black or Japanese Black × Holstein breeds were collected from a local slaughterhouse, transported to the laboratory, washed, and stored in physiological saline. Cumulus oocyte complexes (COCs) were aspirated from small follicles (2–6 mm in diameter), using a 10-mL syringe equipped with a 19-gauge needle^26^.

### Oocyte shipping and oocyte *in vitro* maturation

**Figure 4:**
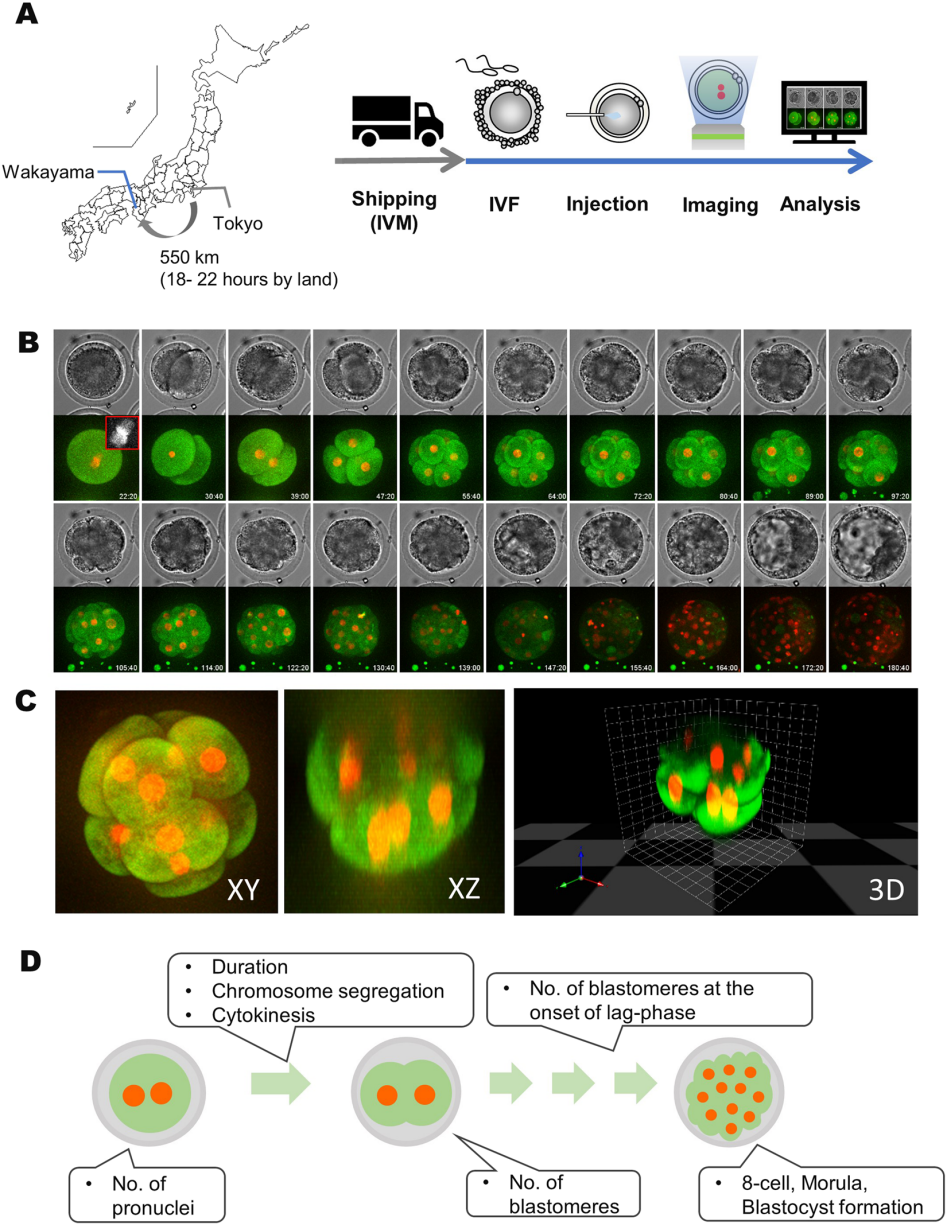
Schematic representation of experimental design. In Tokyo University of Agriculture Technology, Tokyo, Japan, COCs were collected from slaughterhouse-derived ovaries. The collected COCs were shipped while *in vitro* maturing (IVM) to Kindai University, Wakayama, Japan, where we performed live-cell imaging for this study. After arrival, *in vitro* fertilisation (IVF) was conducted for 6 h. mRNAs were injected into oocytes, in which polar bodies were observed, and live-cell imaging was performed for 8 days by CV1000. The obtained images were retrospectively analysed. A map was drawn by JapanPrefecturesMap function in Nippon package of R statistical software (**A**)^30^. 3D live-cell imaging of 369 embryos injected with mRNA encoding histone H2B-mCherry and EGFP-${\rm{\alpha }}$-tubulin was performed from the one-cell to blastocyst stage for 8 days. Maximum intensity projections (MIP) were used for 2D image constrictions. Red and green represent histone H2B-mCherry (nuclei/chromosome) and EGFP-${\rm{\alpha }}$-tubulin (microtubule), respectively (**B**). 2D/3D images of 8-cell embryos were constructed by ImageJ/Fiji and Volocity software. By optimising the imaging conditions, it was possible to obtain images up to the top nuclei/chromosome in the z-axis in lipid-rich bovine embryos (**C**). The number of pronuclei (PN), timing, chromosome segregation, and cytokinesis at first cleavage, number of blastomeres at end of first cleavage, number of blastomeres at the onset of lag-phase were retrospectively observed (**D**).

COCs were shipped to the IVF laboratory, while maturation proceeded *in vitro* (Fig. 4A). The IVM medium was 25 mM HEPES-buffered TCM199 (M199; Gibco, Paisley, Scotland, UK), supplemented with 5% calf serum (CS; Gibco) and 0.1 IU/mL recombinant human follicle-stimulating hormone (FSH) (Follistim; MSD, Tokyo, Japan). COCs were transferred to flat bottom microtubes (TreffLab, Degersheim, Switzerland) with 500 μL of IVM medium (20–40 COCs/tube) and then covered with 300 μL of paraffin oil. The tubes were placed in a cell transport device (Fujihira, Tokyo, Japan), adjusted to 38.5 °C, and shipped. Upon arrival at the IVF laboratory, the tubes were placed in a CO_2_ incubator (Astec, Fukuoka, Japan) at 38.5 °C in a humidified atmosphere of 5% CO_2_ in air, and cultured continuously for up to 22 h of IVM. The developmental competence of oocytes derived from the shipping system was similar to that derived from a conventional IVM system with a CO_2_ incubator (Supplementary Table S8).

### *In vitro* fertilisation (IVF)

IVF was performed as described previously^9^. After 22 h of IVM, ejaculated sperm samples from Japanese Black bulls were thawed and then centrifuged in 3 mL of 90% Percoll solution (GE Healthcare, Uppsala, Sweden) at 750 × *g* for 10 min. After centrifugation, the pellet was re-suspended and centrifuged in 6 mL of sperm washing solution (Brackett and Oliphant solution, BO)^27^, supplemented with 10 mM hypotaurine and 4 U/mL heparin (Novo-Heparin Injection 1000; Aventis Pharma Ltd., Tokyo, Japan), at 550 × *g* for 5 min. Then, the pellet was re-suspended in a sperm washing solution and BO solution, supplemented with 20 mg/mL bovine serum albumin (BSA), to achieve a final concentration of 3 × 10^6^ sperm/mL. This suspension (100 µL) was aliquoted into 35 mm dishes under paraffin oil as fertilisation droplets. COCs were washed twice in BO, supplemented with 10 mg/mL BSA, and cultured in a personal multi-gas incubator (APM-30DR, ASTEC Inc., Fukuoka, Japan) in fertilisation droplets for 6 h at 38.5 °C in a humidified atmosphere of 5% CO_2_ in air.

### Live-cell imaging

Preparation of mRNAs encoding EGFP-α-tubulin and histone H2B-mCherry was described previously^28^. mRNA was synthesised using RiboMAX™ Large Scale RNA Production Systems-T7 (Promega, Madison, USA). The 5′ end of each mRNA was capped using Ribo m7G Cap Analog (Promega). Synthesised RNAs were purified by phenol-chloroform treatment and gel filtration, using a MicroSpin™ G-25 column (GE Healthcare). After insemination, oocytes were completely denuded from cumulus cells and spermatozoa by pipetting with a glass pipette in phenol red-free Charles Rosenkrans 1 with amino acid (CR1aa) medium^29^, supplemented with 5% CS and 0.3% BSA as *in vitro* culture (IVC) medium. mRNA (5 ng/μL each) were injected into ooplasm within four hours of IVF, using a piezo manipulator, in HEPES-buffered CZB medium. The oocytes were transferred to 5-μL droplets of IVC medium on a film bottom dish (Matsunami Glass Ind, LTD., Osaka, Japan). Embryos were imaged three-dimensionally (3D) using a boxed-type confocal laser microscope with a stable incubation chamber (CV1000, Yokogawa Electric Corp., Tokyo, Japan) set at 38.5 °C in 6% CO_2_, 5% O_2_, and 89% N_2_ with saturated humidity. We preliminarily confirmed that a culture volume of 5 μL could support embryo development for 8 days (Supplementary Table S9). To prevent the movement of embryos during imaging, four oocytes were first stuck in each droplet of 2.5 μL protein (BSA and CS)-free CR1aa on the bottom of the dish, and then an additional 2.5 μL of CR1aa containing double the amount of proteins was added by utilizing the property that embryos stuck to the bottom of the dish in macromolecule free media. Images were taken at 10 min intervals for 8 days, using the following laser parameters: EGFP-α-tubulin and histone H2B-mCherry; excitation of 488 nm and 561 nm, emission of 525/50 and 617/73, laser power of 0.05 mW and 0.10 mW, exposure time of 50 msec and 100 msec, gain of 100% and 100%, range of 150 μm and 150 μm, slices of 31 and 31, respectively. The 2D/3D images were constructed by ImageJ/Fiji image analysis platform or Volocity software (PerkinElmer, Inc. Massachusetts, USA) (Figs 1A and 4B,C, and Supplementary Movie S4). The 2D/3D images were retrospectively analysed at several points (Fig. 4D).

### Statistical analysis

All statistical analyses were performed with R statistical software (The R Foundation, version 3.2.4). Unless specified, all functions were used from stats packages. The blastocyst formation rate was compared using a chi-squared test. The timing of first cleavage was compared using a Wilcoxon rank sum test. The variables reflecting timing and blastomere number at first cleavage were identified using a multivariate regression model and multivariate logistic regression model. The variables reflecting blastomere number at the lag-phase were identified using a cumulative logistic regression model with *clm* function in the ordinal package (version 2015.6-28). The cut-off value for timing of first cleavage was determined by *roc* function and *coords* function in the pROC package (version 1.8). A *p*-value < 0.05 was considered significant.

## Electronic supplementary material

Supplementary Movie S1

Supplementary Movie S2

Supplementary Movie S3

Supplementary Movie S4

Supplementary Tables_Figures

## References

[1] Blondin P (2016). Logistics of large scale commercial IVF embryo production. Reproduction, fertility, and development.

[2] Farin PW, Slenning BD, Britt JH (1999). Estimates of pregnancy outcomes based on selection of bovine embryos produced *in vivo* or *in vitro*. Theriogenology.

[3] Petersen BM, Boel M, Montag M, Gardner DK (2016). Development of a generally applicable morphokinetic algorithm capable of predicting the implantation potential of embryos transferred on Day 3. Humanreproduction.

[4] Bo GA, Mapletoft RJ (2013). Evaluation and classification of bovine embryos. Animal Reproduction.

[5] Stringfellow, D. A. & Givens, S. M. Manusal of the International Embryo Transfer Society, 4th ed. (International Embryo Transfer 2010).

[6] Ferraz PA (2016). Factors affecting the success of a large embryo transfer program in Holstein cattle in a commercial herd in the southeast region of the United States. Theriogenology.

[7] Sugimura S, Akai T, Imai K (2017). Selection of viable *in vitro*-fertilized bovine embryos using time-lapse monitoring in microwell culture dishes. The Journal of reproduction and development.

[8] Gardner DK, Meseguer M, Rubio C, Treff NR (2015). Diagnosis of human preimplantation embryo viability. Human reproduction update.

[9] Sugimura S (2012). Promising system for selecting healthy *in vitro*-fertilized embryos in cattle. PloS one.

[10] Yamagata K, Suetsugu R, Wakayama T (2009). Long-term, six-dimensional live-cell imaging for the mouse preimplantation embryo that does not affect full-term development. The Journal of reproduction and development.

[11] Yamagata K, Suetsugu R, Wakayama T (2009). Assessment of chromosomal integrity using a novel live-cell imaging technique in mouse embryos produced by intracytoplasmic sperm injection. Human reproduction.

[12] Li GP (2009). Nicotine combined with okadaic acid or taxol adversely affects bovine oocyte maturation and subsequent embryo development. Fertility and sterility.

[13] Yao G (2016). Developmental potential of clinically discarded human embryos and associated chromosomal analysis. Scientific reports.

[14] Coy P (2008). Oviduct-specific glycoprotein and heparin modulate sperm-zona pellucida interaction during fertilization and contribute to the control of polyspermy. Proceedings of the National Academy of Sciences of the United States of America.

[15] Tsuiko O (2017). Genome stability of bovine *in vivo*-conceived cleavage-stage embryos is higher compared to *in vitro*-produced embryos. Human reproduction.

[16] Sugimura S (2017). Transcriptomic signature of the follicular somatic compartment surrounding an oocyte with high developmental competence. Scientific reports.

[17] Sugimura S (2014). Amphiregulin co-operates with bone morphogenetic protein 15 to increase bovine oocyte developmental competence: effects on gap junction-mediated metabolite supply. Molecular human reproduction.

[18] Ferraz, M. *et al*. Improved bovine embryo production in an oviduct-on-a-chip system: prevention of poly-spermic fertilization and parthenogenic activation. *Lab on a chip* **17**, 905–916 (2017). 10.1039/c6lc01566b 28194463

[19] Mizutani E (2012). Abnormal chromosome segregation at early cleavage is a major cause of the full-term developmental failure of mouse clones. Developmental biology.

[20] Wong CC (2010). Non-invasive imaging of human embryos before embryonic genome activation predicts development to the blastocyst stage. Nature biotechnology.

[21] Booth PJ, Watson TJ, Leese HJ (2007). Prediction of porcine blastocyst formation using morphological, kinetic, and amino acid depletion and appearance criteria determined during the early cleavage of *in vitro*-produced embryos. Biology of reproduction.

[22] Bohrer RC, Coutinho AR, Duggavathi R, Bordignon V (2015). The Incidence of DNA Double-Strand Breaks Is Higher in Late-Cleaving and Less Developmentally Competent Porcine Embryos. Biology of reproduction.

[23] Ganem NJ, Pellman D (2012). Linking abnormal mitosis to the acquisition of DNA damage. The Journal of cell biology.

[24] Kola I, Trounson A, Dawson G, Rogers P (1987). Tripronuclear human oocytes: altered cleavage patterns and subsequent karyotypic analysis of embryos. Biology of reproduction.

[25] Wang ZW (2013). Laser microbeam-induced DNA damage inhibits cell division in fertilized eggs and early embryos. Cell cycle.

[26] Sugimura S (2010). Time-lapse cinematography-compatible polystyrene-based microwell culture system: a novel tool for tracking the development of individual bovine embryos. Biology of reproduction.

[27] Brackett, B. G. & Oliphant, G. Capacitation of rabbit spermatozoa in vitro. *Biology of reproduction* 12, 260–274 (1975). 10.1095/biolreprod12.2.260 1122333

[28] Yamagata K (2005). Noninvasive visualization of molecular events in the mammalian zygote. Genesis.

[29] Rosenkrans CF, Zeng GQ, MCNamara GT, Schoff PK, First NL (1993). Development of bovine embryos *in vitro* as affected by energy substrates. Biology of reproduction.

[30] Team., R. C. R: Foundation for Statistical Computing. *R: A language and environment for statistical computing.* Edn. 3.2.4 (2016).

