# Supplementary material for: Live-cell imaging of nuclear–chromosomal dynamics in bovine in vitro fertilised embryos
Source: Sci Rep. 2018 May 10;8:7460. doi: 10.1038/s41598-018-25698-w (PMC5945782; doi:10.1038/s41598-018-25698-w)
Supplement: Supplementary file 5 — Supplementary Tables_Figures [file 41598_2018_25698_MOESM5_ESM.pdf]

Live-cell imaging of nuclear–chromosomal dynamics in bovine *in vitro* fertilised embryos

Tatsuma Yao[1, 2], Rie Suzuki[1], Natsuki Furuta[1], Yuka Suzuki[1], Kyoko Kabe[1], Mikiko Tokoro[1, 3], Atsushi Sugawara[4, 5], Akira Yajima[4], Tomohiro Nagasawa[4], Satoko Matoba[6], Kazuo Yamagata[1, *], Satoshi Sugimura[4, *]

[1]Faculty of Biology-Oriented Science and Technology (BOST), Kindai University, Wakayama, Japan.

[2]Research and Development Center, Fuso Pharmaceutical Industries, Ltd., Osaka, Japan

[3]Asada Institute for Reproductive Medicine, Asada Ladies Clinic, Aichi, Japan

[4]Department of Biological Production, Tokyo University of Agriculture and Technology, Tokyo, Japan

[5]Institute for Biogenesis Research, University of Hawaii Medical School, Honolulu, Hawaii, USA

[6]Animal Breeding and Reproduction Research Division, NARO Institute of Livestock and Grassland Science, Ibaraki, Japan

*Corresponding authors

Kazuo Yamagata

Faculty of Biology-Oriented Science and Technology (BOST), KINDAI University, 930 Nishimitani, Kinokawa-city, Wakayama 649-6493, Japan

Tel: +81 (736) 77 3888, E-mail: yamagata@waka.kindai.ac.jp

Satoshi Sugimura (Leading corresponding author)

Department of Biological Production, Tokyo University of Agriculture and Technology,

3-5-8 Saiwai-cho, Fuchu, Tokyo 183-8509, Japan
Tel: +81 (42) 367 5819; E-mail: satoshis@cc.tuat.ac.jp

Supplementary Materials and Methods

Embryo transfer and pregnancy diagnosis

The recipients were Japanese Black cows. Embryo transfer were performed on day 8 (day 0 = onset of estrus) by trained technicians. Vitrified single blastocyst on Day 7, which was observed no nuclear /chromosomal abnormality by live-cell imaging, were warmed in a 37°C warming solution. Then, embryo was loaded into 0.25-mL plastic straw and nonsurgically transferred into the uterine horn ipsilateral to the ovary bearing corpus luteum (one embryo per recipient). Cooling and embryo warming were performed using the Cryotop methodology for human embryo vitrification described by Kuwayama et.[1]. Equilibration, vitrification, warming, dilution, and washing solutions were provided in the Vitrification Kit (VT-101 and VT-102; Kitazato Biopharma). Pregnancy was diagnosed by observation of a fetus with a detected heartbeat in the intraluminal uterine fluid and embryonic membrane using the ultrasonography on days 31 and 45 (after days 23 and 37 of embryo transfer, respectively). The recipients were housed in a loose barn and fed based on the and managed in the Japanese Feeding Standard for Beef Cattle (National Agricultural Research Organization, 2008).

Effect of okadaic acid on bovine embryo development

For inducing mitotic abnormality, putative zygote after *in vitro* fertilization or cumulus oocyte complexes (COCs) were treated with culture medium including 0.02, 0.04, or 0.05 μM okadaic acid (Wako, Osaka, Japan) for 8 days or 22 hours, respectively. Concentration of okadaic acid was according to previous study[2]. Embryos injected with mRNA encoding histone H2B-mCherry were analysed in vitro development by live-cell imaging for 8 days (Supplementary Fig. S1 and Supplementary Movie S3).

Karyotyping of blastocysts

Chromosome samples of embryos were prepared as previously described by Sugimura et al.[3]. Blastocysts were cultured for 14–17 h in CR1aa supplemented with 5% CS and 60 ng/ml vinblastine sulfate (Wako Pure Chemical Industries, Osaka, Japan). Blastocysts were then washed and incubated in 1% (w/v) sodium citrate solution for 15 min and fixed by pouring 0.02 ml acetic methanol (1:1 (v/v) acetic acid/methanol) into 0.4 ml of a hypotonic solution of sodium citrate. A blastocyst was placed on a glass slide in air with saturated humidity, immediately covered with a very small droplet of acetic acid to separate each cell, and then re-fixed with acetic alcohol (1:3 (v/v) acetic acid/methanol). After complete drying, chromosome samples were stained with 2% (w/w) Giemsa in distilled water (Merck KGaA, Darmstadt, Germany) for 10 min. Only cell nuclei that were intact and non-overlapping were analyzed. For each embryo, all analyzable metaphases were examined under oil-immersion bright-field microscopy at a magnification of 1000×(100×10) to determine the chromosome number. Embryos in which all nuclei analyzed contained two sets of chromosomes (2n =60) or one set of chromosomes (n= 30) were scored as diploid or haploid, respectively, and those in which all nuclei contained 3n, 4n or ≥5n were considered polyploid. Embryos containing a mixture of diploid cells and cells with more or fewer than two sets of chromosomes were considered mixoploid.

Reference

1. Kuwayama, M., Vajta, G., Kato, O. & Leibo, S.P. Highly efficient vitrification method for cryopreservation of human oocytes. *Reproductive biomedicine online* **11**, 300-308 (2005).
2. Li, G.P. et al. Nicotine combined with okadaic acid or taxol adversely affects bovine oocyte maturation and subsequent embryo development. *Fertility and sterility* **92**, 798-805 (2009).

3. Sugimura, S. et al. Promising system for selecting healthy in vitro-fertilized embryos in cattle. *PloS one* **7**, e36627 (2012).

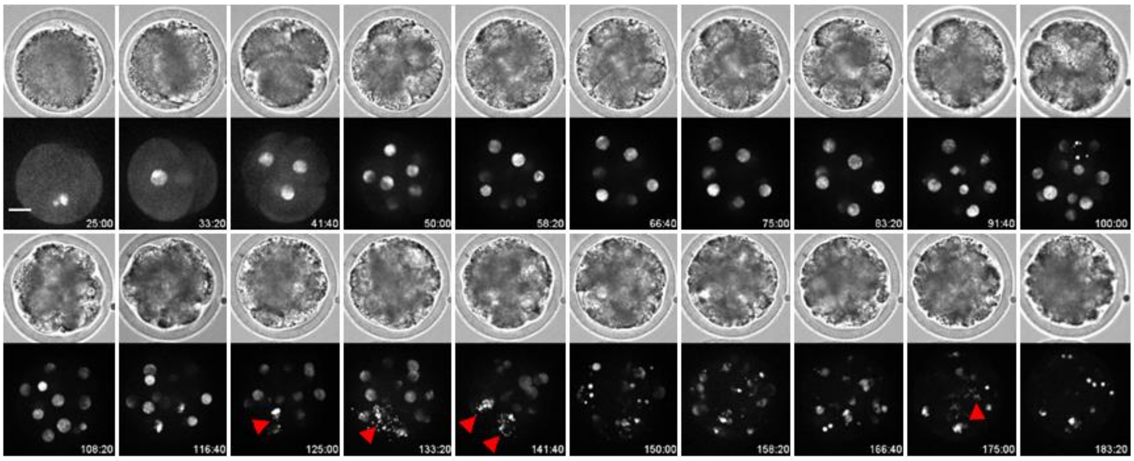

Supplementary Figure S1. Typical pattern of chromosome dynamics of bovine embryo treated with 0.04 μM okadaic acid during *in vitro* culture. The signals in the lower panels represent histone H2B-mCherry. Red arrows indicate sever abnormal chromosome segregation.

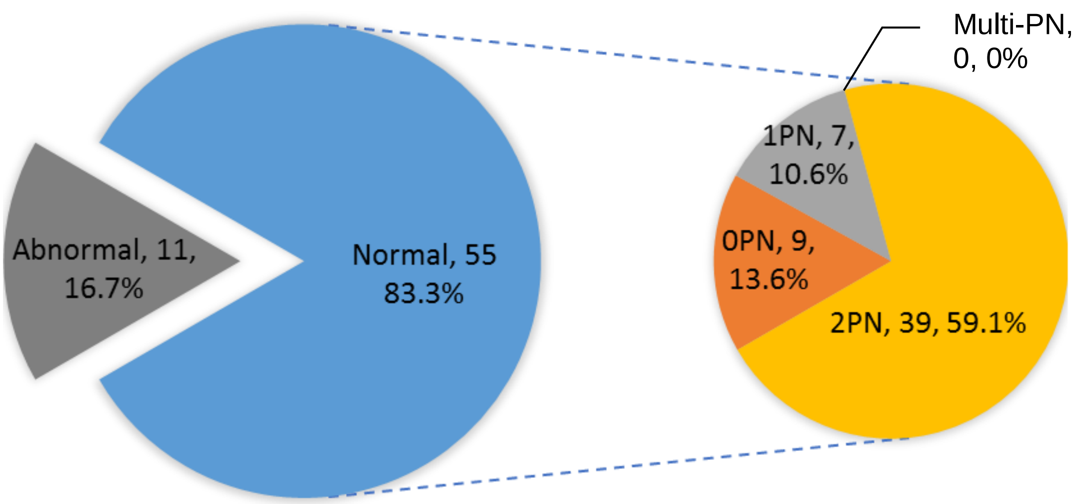

Supplementary Figure S2. The proportion of blastocysts derived from zygotes with no pronucleus (0 PN), one pronucleus (1 PN), two pronuclei (2 PN), and more than three pronuclei (multi-PN) graded as normal based on blastomere numbers at end of first cleavage. Blastocysts derived from embryos that accomplished two blastomeres at the end of first cleavage were defined as normal.

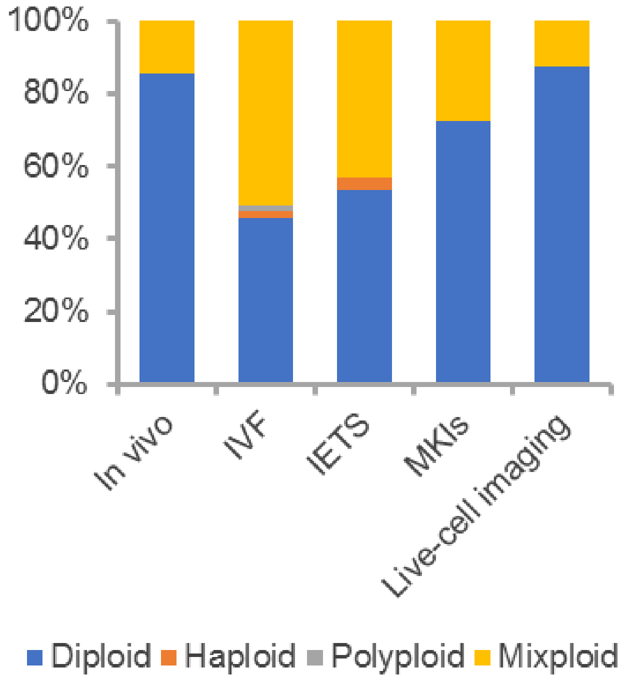

Supplementary Figure S3. Ploidy in *in vivo* and *in vitro* derived blastocyst. *In vivo* derived blastocysts (in vivo: N=14), *in vitro* derived blastocysts (IVF: N=61) and IVF blastocysts selected by criteria of International Embryo Transfer Society (IETS: N=28), morphokinetics indicators (MKIs: N=29) and live-cell imaging (Live-cell imaging: N=8) were analyzed by karyotyping with Giemsa stain. According to IETS criteria, code 1 and 2 were selected. MKIs selected blastocysts derived from embryos that accomplished the first cleavage within 32.3 h post-insemination, 2 blastomeres at the end of first cleavage, and ≧6 blastomeres at the onset of lag-phase. In Live-cell imaging, embryos accomplished normal first cleave with two pronuclei and two blastomeres and without abnormal chromosome segregation and cytokinesis and lag-phase with ≧ 6 blastomeres were selected.

Supplementary Table S1. Effect of okadaic acid on in vitro bovine embryo development*

Okadaic acid (μM)	Treated period	Analyzed	First cleavage (%)	≧8cell (%)	≧Morula (%)	≧Blastocyst (%)
0.00	-	23	22 (95.7)	15 (65.2)	13 (56.5)	12 (52.2)
0.02	IVC	25	23 (92.0)	14 (56.0)	6 (24.0)	0 (0.0)
0.04	IVC	27	26 (96.3)	13 (48.1)	2 (7.4)	0 (0.0)
0.05	IVM	19	18 (94.7)	4 (21.1)	3 (15.8)	2 (10.5)

*Embryo development was analysed by live-cell imaging for 8 days.

Supplementary Table S2. Effect of okadaic acid on abnormal mitosis during first cleavage to morula*

Okadaic acid (μM)	Treated period	Abnormal cytokinesis at first cleavage (%)	Multi-division at first cleavage (%)	ACS at first cleavage (%)	ACS up to morula (%)	Severe ACS up to morula (%)
0.00	-	1/22 (4.5)	3/22 (13.6)	7/22 (31.8)	10/22 (45.5)	9/22 (40.9)
0.02	IVC	3/23 (13.0)	4/23 (17.4)	9/23 (39.1)	22/23 (95.7)	19/23 (82.6)
0.04	IVC	4/26 (15.4)	6/26 (23.1)	8/26 (30.8)	24/26 (92.3)	20/26 (76.9)
0.05	IVM	6/18 (33.3)	4/18 (22.2)	7/18 (38.9)	15/18 (83.3)	10/18 (55.6)

*Embryo development was analysed by live-cell imaging for 8 days.

Supplementary Table S3. Multivariate analysis of variables reflecting blastocyst development

Variables	*Estimate*[a]	*SEM*[b]	*ORadj*[c]	*95% CI*[d]	*P-value*
(Intercept)	-0.84	0.19	-	-	< 0.001
0 PN [Yes]	-0.48	0.42	0.62	0.27 - 1.40	0.251
1 PN [Yes]	-1.09	0.44	0.34	0.14 - 0.80	0.014
Multi-PN [Yes]	-0.16	0.52	0.85	0.31 - 2.36	0.752
ACS [Yes]	-1.43	0.43	0.24	0.10 - 0.56	< 0.001
Abnormal cytokinesis	-1.49	1.05	0.23	0.03 - 1.75	0.155
Multi-division	-0.30	0.42	0.74	0.33 – 1.69	0.476

Supplementary Table S4. Multivariate analysis of variables reflecting timing of first cleavage

Variables	*Estimate*[a]	*SEM*[b]	*95% CI*[c]	*VIF*[d]	*P-value*
(Intercept)	31.95	0.60	30.78 - 33.12	-	< 0.001
0 PN [Yes]	2.42	1.19	0.09 - 4.76	1.10	0.042
1 PN [Yes]	4.41	1.03	2.39 - 6.44	1.11	< 0.001
Multi-PN [Yes]	-0.40	1.16	-2.68 - 1.89	1.14	0.734
ACS [Yes]	5.33	0.87	3.63 - 7.05	1.07	< 0.001

[a] Coefficient estimate of multivariate analysis.

[b] Standard error of estimate.

[c] 95% confidence interval.

[d] Variance inflation factor

Supplementary Table S5. Multivariate analysis of variables reflecting multiple blastomeres at end of first cleavage

Variables	*Estimate*[a]	*SEM*[b]	*ORadj*[c]	*95% CI*[d]	*P-value*
(Intercept)	-1.96	0.24	-	-	< 0.001
0 PN [Yes]	0.64	0.41	1.91	0.86 - 4.22	0.112
1 PN [Yes]	-0.53	0.48	0.59	0.23 - 1.50	0.267
Multi-PN [Yes]	2.67	0.37	14.45	7.01 - 29.82	< 0.001
ACS [Yes]	0.33	0.31	1.39	0.75 - 2.57	0.293

[a] Coefficient estimate of multivariate analysis.

[b] Standard error of estimate.

[c] Adjusted odds ratio

[d] 95% confidence interval.

Supplementary Table S6. Multivariate analysis of variables reflecting number of blastomere at onset of lag-phase

Variables	*Estimate*[a]	*SEM*[b]	*ORadj*[c]	*95% CI*[d]	*P-value*
3-5 | 6-8	-0.51	0.19	0.60	0.41 - 0.88	-
6-8 | 9-16	2.26	0.27	9.61	5.71 - 16.19	-
0 PN [Yes]	0.49	0.40	1.64	0.75 - 3.60	0.219
1 PN [Yes]	-0.18	0.37	0.84	0.41 - 1.72	0.625
Multi-PN [Yes]	1.54	0.47	4.67	1.88 - 11.62	0.001
ACS [Yes]	-0.90	0.38	0.41	0.19 - 0.86	0.018

[a] Coefficient estimate of multivariate analysis.

[b] Standard error of estimate.

[c] Adjusted odds ratio

[d] 95% confidence interval.

Supplementary Table S7. Frequency and subsequent developmental competence of oocytes with different number of pronuclei (PN) and with/without abnormal chromosomal segregation (ACS) to blastocyst stage

No. of PN	ACS	No. of observed oocytes	No. of observed blastocysts (%)
0	Yes	7	0 (0)
	No	43	9 (20.9)
	Total	50	9 (18.0)
1	Yes	18	0 (0)
	No	54	7 (13.0)
	Total	72	7 (9.7)
2	Yes	57	5 (8.8)
	No	135	38 (28.1)
	Total	192	43 (22.4)
≥3 (Multi)	Yes	31	2 (6.5)
	No	24	5 (20.8)
	Total	55	7 (12.7)

Supplementary Table S8. Effect of cell transporter during *in vitro* maturation (IVM) on in vitro bovine embryo development.

Instrument	IVM system	No. of cultured oocytes (replicates)	Percentage of embryos (mean ± SD%)			
			Cleavage at day 2		Developed to blastocyst stage at day 8	
			Total	4-cell <	Total	Expanded stage
CO_2 incubator	Petri dish	105 (5)	71.8 ± 13.7	57.6 ± 14.0	23.4 ± 9.5	13.0 ± 6.7
Cell transporter	Microtube	136 (5)	70.1 ± 10.3	56.8 ± 10.1	29.0 ± 11.9	18.3 ± 1.5

Supplementary Table S9. Effect of culture volume on *in vitro* bovine embryo development

Culture volume (µl)	No. of cultured oocytes (replicates)	Percentage of embryos (mean ± SD%)			
		Cleavage at day 2		Developed to blastocyst stage at day 8	
		Total	4-cells	Total	Expanded stage
5	60 (5)	70.0 ± 9.5	58.3 ± 4.6	33.3 ± 5.9	25.0 ± 8.3
10	60 (5)	80.0 ± 7.5	65.0 ± 3.7	33.3 ± 8.3	26.7 ± 10.9
20	60 (5)	75.0 ± 13.2	60.0 ± 10.9	41.7 ± 5.9	38.3 ± 4.6

Supplementary Movie S1. Colour doppler ultrasound image of the pregnancy status on 45 days in a recipient transferred live-cell imaged bovine IVF embryo. The transferred embryo was not observed nuclear / chromosomal abnormality such as abnormal chromosome segregation and abnormal number of pronuclei.

Supplementary Movie S2. Three dimensional live-cell imaging of bovine IVF embryos directly cleaved from one cell to four blastomeres (multi-divistion). Images were taken at 10 min intervals for 8 days. Red and green represent histone H2B-mCherry (nuclei/chromosome) and EGFP-α-tubulin (microtubule), respectively.

Supplementary Movie S3. Three dimensional live-cell imaging of bovine IVF embryos treated with 0.04 μM okadaic acid during IVC. Images were taken at 10 min intervals for 8 days. Gary signals represent histone H2B-mCherry (nuclei/chromosome).

Supplementary Movie S4. Three dimensional live-cell imaging of normal cleaved bovine IVF embryos with two pronuclei and normal chromosome segregation. Images were taken at 10 min intervals for 8 days. Red and green represent histone H2B-mCherry (nuclei/chromosome) and EGFP-α-tubulin (microtubule), respectively.
